# Supplementary figures and images for: Noninvasive Mapping of Angiotensin Converting Enzyme-2 in Pigeons Using Micro Positron Emission Tomography
Source: Life (Basel). 2022 May 26;12(6):793. doi: 10.3390/life12060793 (PMC9224634; doi:10.3390/life12060793)

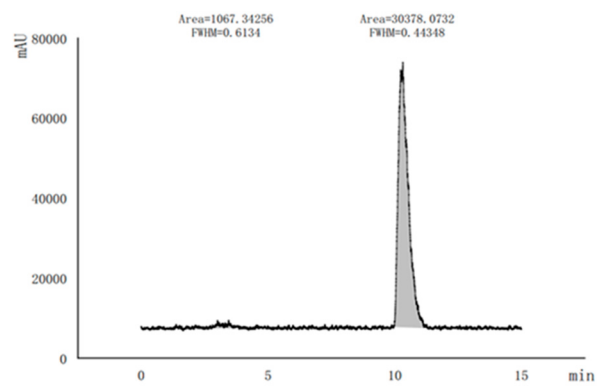

**Figure S1.** Radio-HPLC of  $^{68}\text{Ga}$ -HZ20.

Supplement: Supplementary file 1 [file life-12-00793-s001.zip › life-1670083-supplementary.pdf]
